# Dynamic characteristics of coal gangue subgrade filler under traffic load based on the hysteretic curves

**DOI:** 10.1038/s41598-024-62391-7

**Published:** 2024-05-21

**Authors:** Lian-Zhi Zhang, Cun-Bo Kang, Zong-Tang Zhang

**Affiliations:** 1 Geospatial Survey and Monitoring Institute of Hunan Province, Changsha, 410000 People’s Republic of China; 2 Hunan Provincial Key Laboratory of Geotechnical Engineering for Stability Control and Health Monitoring, Hunan University of Science and Technology, Xiangtan, 411201 People’s Republic of China

**Keywords:** Subgrade, Dynamic triaxial, Coal gangue, Traffic loading, Hysteretic curve, Environmental sciences, Solid Earth sciences

## Abstract

Traffic cyclic loading is the key factor that leads to the deterioration of the long-term service behavior of subgrade. A series of cyclic triaxial tests was carried out by the large-scale dynamic and static triaxial apparatus (LSDSTA) to study the dynamic behaviors of coal gangue subgrade filler (CGSF) under multi-step cyclic loading using the morphological characteristics of hysteretic curves (MCHC). MCHC was quantitatively characterized by four parameters, i.e., the unclosed degree (*ε*_*phl*_*)*, inclination of long axis degree (*k*_*hl*_), area (*S*_*hl*_) and fullness degree (*α*_*hl*_). With the increase of dynamic strain, *ε*_*phl*_ increases exponentially. *k*_*hl*_ of the coal gangue sample first decreases and then shows an increasing trend with the increasing dynamic strain. The values of *S*_*hl*_ are close to each other, and the energy dissipation in the sample is small. However, with the increase of dynamic strain, the specimen failure degree is increased, *S*_*hl*_ increases exponentially, and the damping ratio increases. With the increase of dynamic strain, *α*_*hl*_ increases approximately linearly. Confining pressure has a certain effect on the four parameters. There parameters can be recommended and used for quantitative analysis the dynamic behaviors of subgrade filler under traffic cyclic loading.

## Introduction

In order to rationally utilize resources and save costs, nearby materials in the surrounding quarries should be used for subgrade filler in road engineering, while the construction of railways and highways has a huge demand for filling materials. Predatory mining in quarries has a significant impact on the surrounding environment and ecology. If coal gangue is used for the construction of subgrade, it will generate significant economic, social, and environmental benefits.

Coal gangue is a type of hard black rock with low carbon content, which is separated during mining and washing processes and becomes waste^1–3^. The massive use of coal gangue waste has always been a hot topic concerned by many scholars^4–7^. The physical and chemical characteristics of coal gangue are similar to natural gravel, so coal gangue can be broken into coarse aggregate, which can decrease the exploitation of natural gravel, but also avoid a series of hazards caused by it^8^. Hence, the study on coal gangue aggregate has been focused by many scholars in recent years, such as^9–12^. Comparatively, the demand of coal gangue as subgrade filler is greater, and the vast majority of existing coal gangue is suitable as a geotechnical filler^13–15^. Therefore, the effective utilization of coal gangue as subgrade filling has great practical significance.

Coal gangue used as subgrade filler is an important measure to solve the problem of coal gangue accumulation. In order to prevent the secondary environmental pollution caused by the coal gangue subgrade, a mature construction method, i.e., lay clay on the surface of the coal gangue subgrade, has been widely used^16,17^. Then, coal gangue is widely used as subgrade filler with the rapid development of traffic construction in China. E.g., Jining-Yutai (Shandong-Jiangsu border) section of Ji Xu expressway, K127 + 760.000 section of Xu-Huai expressway^18^, Wangshan section of S102 in Anhui Province, Pinglin expressway, Qingxu section of 307 national highway, Xingtai-Hebei and Shanxi boundary of Xingfen expressway^16^. Therefore, the application of coal gangue as subgrade filler is feasible and extensive. The consumption of coal gangue used as subgrade filler is tremendous, which can effectively solve the problem of coal gangue accumulation.

There have been some researches on using coal gangue as subgrade filler materials. Jiao et al.^19^ the inorganic salt content and heavy metals of coal gangue used in Xing Fen expressway were analyzed through laboratory soak test and leaching test, groundwater in Xing Fen highway was captured, detected, evaluated and its quality was good or excellent, and then coal gangue as a new filler can be applied to coastal highway roadbed. Li et al.^20^ focused on the utilization of coal gangue aggregate in railway engineering for coal transportation passage. Chen et al.^21^ studied the effect of the compactive effort and initial particle gradations on the particle size distribution of mineral waste slag based on screening tests, and analyzed the effects of different factors such as the compactive effort, moisture content, coarse grain content (CGC, mass proportion of particles with sizes greater than 5 mm), and forming methods on the engineering properties of mineral waste slag to determine the reasonable roadbed construction parameters. Zhang et al.^1^ investigated the permanent deformation and its unified model of coal gangue subgrade filler under traffic cyclic loading. Tang et al.^22^ studied the residual deformation of coal gangue subgrade filler under multi-vibration cyclic loading. In addition, the effect of gradation on the compaction and strength characteristics of coal gangue subgrade filler was also studied^17,23,24^. As mentioned above, coal gangue is widely used as subgrade filler. The coal gangue subgrade is affected by the traffic dynamic loading in the actual environment, while there are few researches reporting the dynamic behaviors of coal gangue, especially considering coarse particles, used as subgrade filler under traffic cyclic loading.

Hysteresis curve morphological characteristics can characterize the important dynamic behaviors of the specimen, such as dynamic deformation, viscosity, stiffness, and energy loss. The study of dynamic characteristics of coal gangue subgrade filler based on the hysteretic curves is of great significance for understanding the dynamic behaviors of actual subgrade engineering under traffic load. Hence, a series of cyclic triaxial tests was carried out by the LSDSTA (large-scale dynamic and static triaxial apparatus) to research the dynamic properties of CGSF, i.e., coal gangue subgrade filler (the maximum particle size is 60 mm) under multi-step cyclic loading using the MCHC (morphological characteristics of hysteretic curves). MCHC was quantitatively characterized by four parameters, i.e., ${\varepsilon }_{phl}$, ${k}_{hl}$, ${S}_{hl}$, and ${\alpha }_{hl}$, and then the residual strain, elastic properties, energy dissipation, and viscosity of CGSF under cyclic loading were analyzed to study the dynamic behaviors of CGSF.

## Laboratory testing program

### Tested materials and apparatus

**Figure 1:**
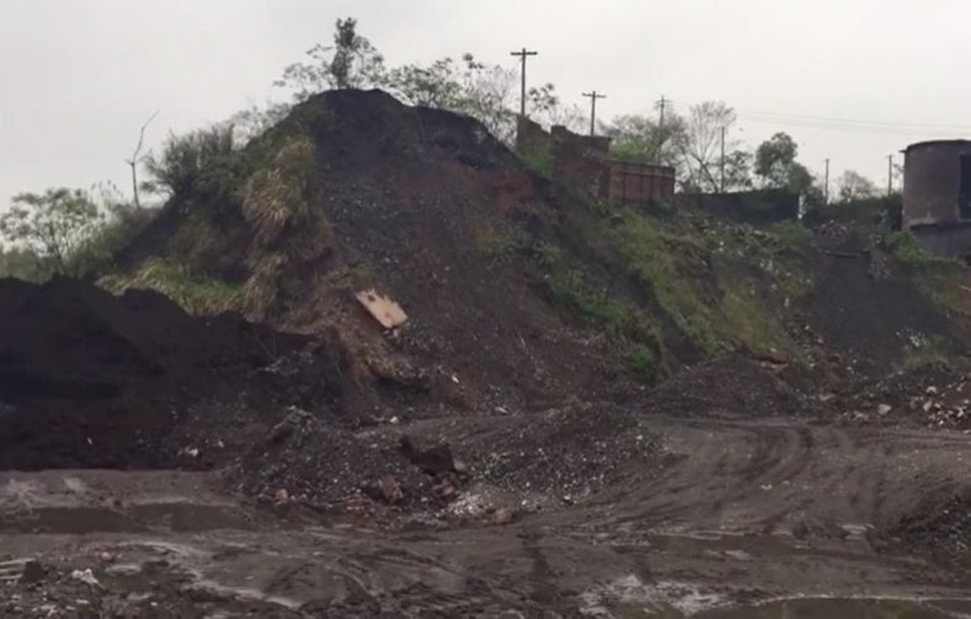
Coal gangue particles of each group after sieving tests.

The original tested materials of the specimens were crushed coal gangue, which were collected from a coal mine in Xiangtan city (as shown in Fig. 1). It should be noted that, the maximum particle size allowed by the test equipment in this test does not exceed 60 mm. Considering that the specimens in this test were prepared manually, CGSF with particle size greater than 60 mm were removed. Coal gangue particles are angular and sharp, rough surface, irregular shape and hard. The CGSF was dried to constant weight in an oven at 105 ℃–110 ℃ (more than 24 h), and then the standard sieve tests with aperture sizes of 60, 40, 20, 10, 5, 2, 0.5 and 0.075 mm were carried out.

**Figure 2:**
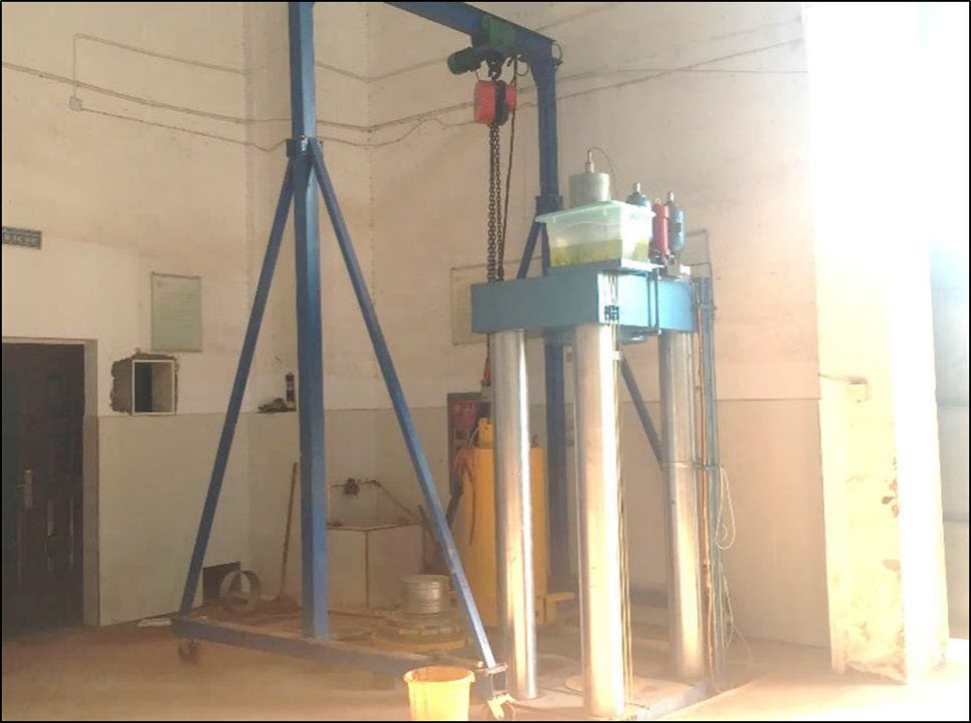
The large-scale dynamic and static triaxial apparatus.

LSDSTA was used in this experiment, which is displayed in Fig. 2. In this apparatus, the mould is used to prepare the sample. Then the prepared sample is pushed into the triaxial pressure cover, and the testing machine control system that can automatically collect axial and volumetric deformation as well as pore pressure is used to achieve cyclic loading. The parameters of LSDSTA are displayed in Table 1. The sample size allowed by LSDSTA is $D$ = 300 mm and $H$ = 600 mm. The ratio between the triaxial specimen diameter and the maximum particle size should not be smaller than 5^25–27^, therefore, the maximum particle size allowed by LSDSTA in this test does not greater than 60 mm, and then the effect of specimen size can be neglected. In addition, the large-scale triaxial tests and the same specimen size were widely used in the previous studies, e.g., Cai et al.^26^ and Leng et al.^27^, which guarantees there liability of this apparatus.

**Table 1:** The parameters of LSDSTA.

Parameters	Values
Specimen size (${\text{mm}}$)	$\phi { }300 \times 600$
Maximum axial static loading (${\text{kN}}$)	$\le 1500$
Maximum axial dynamic loading (${\text{kN}}$)	$\le 700$
Maximum axial displacement (${\text{mm}}$)	$\le 90$
Maximum confining pressure (${\text{MPa}}$)	$\le 3$
Maximum pore pressure (${\text{MPa}}$)	$\le 3$
Maximum vibration frequency (${\text{Hz}}$)	$\le 5$
Loading patterns	Half-sine wave, square wave, oblique wave, triangular wave, random wave and combined wave
Control system	Stress controlled loading, strain controlled loading and displacement controlled loading

### Experimental design

Considering that the fractal model gradation equation (FMGE) has a clear physical meaning and only one parameter facilitates the discussion of results. Therefore, the method of artificial preparation specimens is used according to FMGE in this test, and the FMGE is defined as^28–31^

1 $$P_{i} = \left( {\frac{{d_{i} }}{{d_{max} }}} \right)^{{3 - D_{f} }} \times 100\%$$

where $D_{f}$ represents the fractal dimension, $d_{i}$ presents the particle size (mm), $P_{i}$ denotes the cumulative mass percentage with particle size less than $d_{i}$ (%), and $d_{max}$ indicates the maximum particle size (mm).

**Figure 3:**
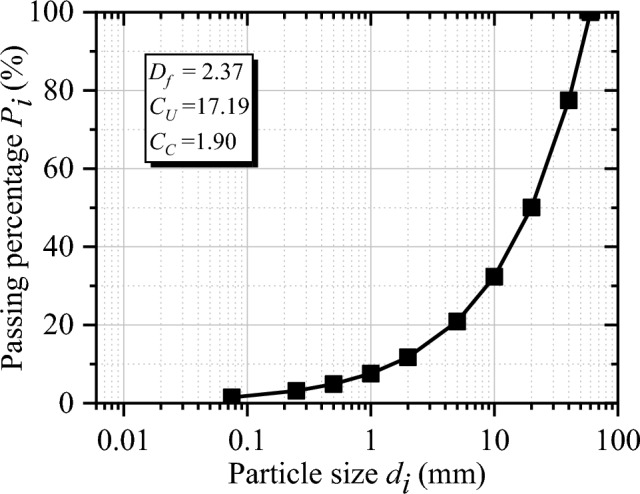
Particle size distribution of samples.

The specimen gradation (i.e., particle size distribution) curve with $D_{f}$ = 2.37 is shown in Fig. 3. The coefficient of uniformity ($C_{U}$) and the coefficient of curvature ($C_{C}$) are 17.19 and 1.90 respectively. Cylindrical specimen with $D$ = 300 mm and $H$ = 600 mm was utilized in this test, note that $D$ and $H$ are the diameter and height of the specimen, respectively. According to the Chinese Standard of Soils for Highway Engineering^32^, the maximum dry density (2.075 ${\text{ g}}/{\text{cm}}^{3}$) of the specimen is obtained.

Compaction degree, which was widely used for triaxial test and situ construction^33,34^, is calculated as follows:

2 $$K = \frac{\rho }{{\rho_{max} }} \times 100$$

where $\rho_{max}$ denotes the maximum dry density (${\text{g}}/{\text{cm}}^{3}$), and $\rho$ is the dry density of specimen (${\text{g}}/{\text{cm}}^{3}$), and $K$ represents the compaction degree (%).

In this test, the compaction degree is used to control the preparation of specimens, and the compaction degree of specimen is 93%. In addition, the compaction degree meets the requirements of the road base and subbase materials.

**Figure 4:**
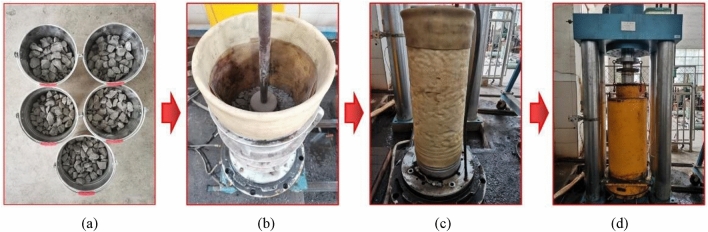
The process of the large triaxial tests: (**a**) CGSF required for a whole specimen; (**b**) compacting in a mould; (**c**) the prepared specimen; (**d**) enclosing the triaxial pressure cover.

Following the Chinese Standard of Soils for Highway Engineering^32^, to ensure the consistency of the specimen quality, a standard routine for the experimental procedure of the sample was used and the dry soil method was used to prepare samples. Figure 4 shows the process of the large triaxial tests, and the experimental steps are:

1. The drying CGSF should be well mixed according to the designed gradation curve (as shown in Fig. 3).
2. The specimen was compacted with a compaction hammer in a mould in five layers (as shown in Fig. 4b). Thickness of each individual layer was 120 mm, and the mass of added CGSF in each layer was near to 16 kg. Before placing CGSF for the next layer, the surface of the previously compacted layer was scraped to a depth of about 20 mm to guarantee well interlocking between the vertically adjacent layers. The target compaction degree of the CGSF specimen was reached by controlling the thickness of each individual layer and the mass of added CGSF.
3. After compaction, a rubber membrane was used to enclose the specimen, and the top and bottom of the specimen were tied with the rubber ropes. Figure 4c shows the prepared specimen.
4. The specimen was put in the triaxial pressure cover, as shown in Fig. 4d. All of the specimens were saturated by back pressure before loading. The specimens were considered completely saturated when the pore pressure coefficient $B$ was larger than 0.95.
5. After that, the required effective confining pressure was applied to the specimen to complete the isotropic consolidation.
6. Carry out cyclic triaxial tests using the large-scale dynamic and static triaxial apparatus.

The research shows that the traffic loading is different from the sine wave, but very similar to the half-sine wave^35^. Therefore, the half-sine wave was used to simulate the traffic cyclic dynamic loading. The dynamic loading frequency was chosen as 1 Hz in these tests, which was also used in^27,35^. This paper mainly focuses on the evolution of hysteretic curves of CGSF under different confining pressures, hence, the confining pressures (i.e., effective consolidation stress), $\sigma_{3}{\prime}$, of 50, 100, 150, and 200 ${\text{kPa}}$ were selected in this test. Note that, three groups of parallel tests were carried out at the same time under each test condition, and first group was selected for key analysis. In order to ensure the uniformity of the specimen and eliminate the dispersion of the test results, step loading method was utilized in the dynamic triaxial test. Each step loading should be loaded 10 times, and the sixth complete loading process was mainly analyzed, which was also used in^36^ and^37^.

Cai et al.^38^ defined cyclic stress ratio ($CSR$) as

3 $$CSR = \frac{{{\Delta }q_{cyc} }}{{\sigma_{3}{\prime} }}$$

**Figure 5:**
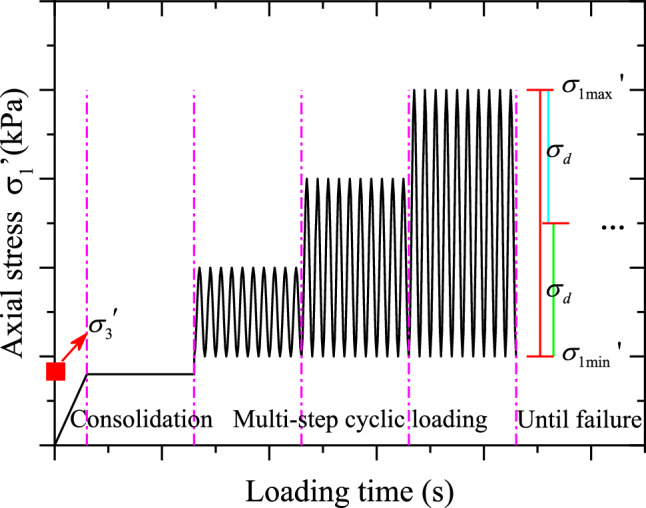
Loading process of specimen under cyclic loading.

The $CSR$ was taken as 1, 1.5, 2, …, until the specimen was destroyed, i.e., the test loading stopped when the accumulated strain reached 15%^36^. The loading process in this test was shown in Fig. 5. According to Indraratna et al.^39^ and Leng et al.^27^, the relevant experimental parameters were defined as follows

4 $$q_{max,cyc} = \sigma_{1max}^{\prime } - \sigma_{3}^{\prime }$$

5 $$q_{min,cyc} = \sigma_{1min}^{\prime } - \sigma_{3}^{\prime }$$

6 $${\Delta }q_{cyc} = q_{max,cyc} - q_{min,cyc}$$

7 $$\sigma_{d} = {\Delta }q_{cyc} /2$$

where $\sigma_{1}^{\prime }$ represents the axial stress during cyclic loading (${\text{kPa}}$); $\sigma_{3}^{\prime }$ denotes the effective confining pressures (${\text{kPa}}$); $q_{max,cyc}$ indicates the maximum cyclic deviatoric stress under different dynamic stress amplitudes (${\text{kPa}}$); $q_{min,cyc}$ presents the minimum cyclic deviatoric stress under different dynamic stress amplitudes (${\text{kPa}}$); $\sigma_{d}$ denotes dynamic stress (${\text{kPa}}$).

**Figure 6:**
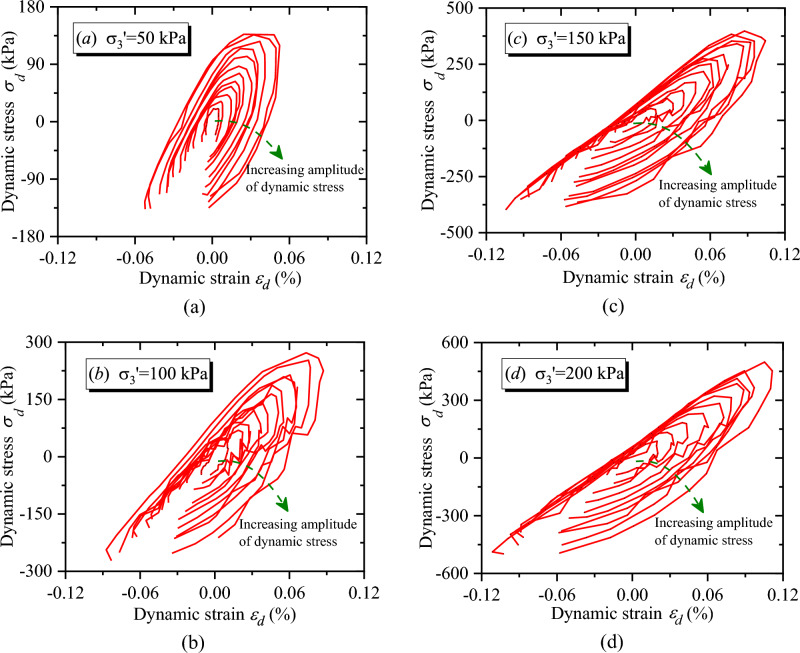
The hysteresis loops of CGSF: (**a**) $\sigma_{3}^{\prime } = 50{\text{ kPa}}$, (**b**) $\sigma_{3}^{\prime } = 100{\text{ kPa}}$, (**c**) $\sigma_{3}^{\prime } = 150{\text{ kPa}}$, (**d**) $\sigma_{3}^{\prime } = { }200{\text{ kPa}}.$

As mentioned above, $CSR$ defined in Eq. (3) was used for stress controlled step loading test. Then, 10 numbers cyclic loading was used for each step loading, and the hysteresis loop under the 6th number cyclic loading of each step loading was selected for research. Hysteretic curve refers to the relationship curve between dynamic stress and dynamic strain with the coordinate origin as the center in one cyclic loading, and Fig. 6 displays the hysteresis curve of CGSF under different confining pressures. It can be seen from Fig. 6 that the hysteresis curve of CGSF under cyclic loading is approximately oval, and the hysteresis loop is not smooth. Furthermore, with the increase of dynamic strain and confining pressure, the gap between loading and unloading, the overall inclination angle, the enclosed area and the flattening degree of hysteresis curve have undergone great variation. Therefore, in order to accurately analyze the variation of hysteretic curve under cyclic loading, the unclosed degree ($\varepsilon_{phl}$), the inclination of long axis degree ($k_{hl}$), the area ($S_{hl}$) and the fullness degree ($\alpha_{hl}$) for hysteretic curve were introduced to quantitatively investigate the evolution of MCHC of CGSF.

## Quantitative analysis of MCHC

The hysteresis curve, also known as hysteresis loop, reflects the shape of dynamic stress- dynamic strain at different times in the process of unloading and reloading. Theoretically, the hysteresis curve is the curve of the forward helix along the transverse axis. In order to better study the periodic relationship between displacement and load, the center of each curve is translated to the coordinate origin. Further, the evolution of dynamic stress- dynamic strain can be fully understood by tracking the variation of hysteresis curve^40^.

Hysteretic curve is the essential reflection of dynamic characteristics for materials under cyclic loading, and is the core for building the dynamic constitutive model of materials. MCHC can reflect the macroscopic mechanical characteristics of materials^37^. Hence, the research on the MCHC has important theoretical and practical significance. Referring to the above studies, MCHC of CGSF under cyclic loading was made a quantitative analysis in this paper.

### Analysis of the unclosed degree for hysteretic curve

**Figure 7:**
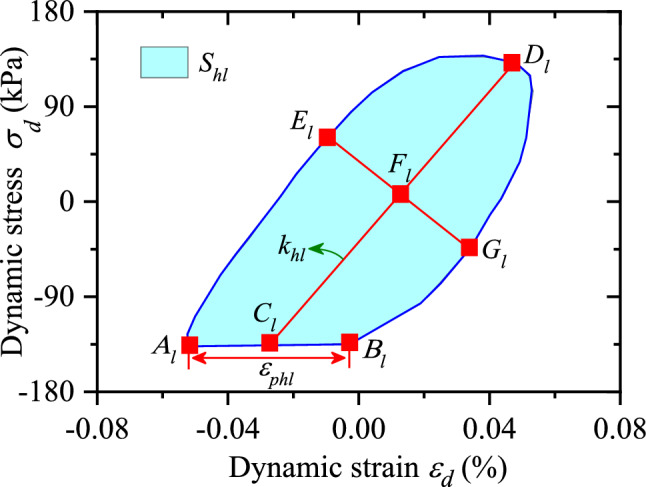
A schematic diagram for the calculation of MCHC.

Figure 6 illustrates the hysteresis curve of CGSF under different confining pressures. As observed in Fig. 6, the hysteresis curve of CGSF under cyclic loading is not closed. The starting point of hysteresis curve in Fig. 6 is at the beginning of loading under cyclic loading in the test, and the end point of hysteresis curve is at the finish of unloading under cyclic loading. The difference between the strain corresponding to the loading beginning point (as shown in point $A_{l}$ in Fig. 7) and the unloading finish point (as indicated in point $B_{l}$. in Fig. 7) in one cycle is used to characterize the unclosed degree of the hysteresis curve (as demonstrated in Fig. 7, a schematic diagram f quantitative calculation of MCHC when the confining pressure was 50 ${\text{kPa}}$ and the 60th step loading is taken as an example), which is defined as

8 $$\varepsilon_{phl} = \left| {\varepsilon_{pe} - \varepsilon_{pb} } \right|$$

where $\varepsilon_{phl}$ indicates the unclosed degree of hysteresis curve, $\varepsilon_{pe}$ denotes the unloading finish point (as indicated in point $B_{l}$) in one cyclic loading, and $\varepsilon_{pb}$ represents the loading beginning point (as displayed in point $A_{l}$) in one cyclic loading.

The unclosed degree of hysteretic curve characterizes the residual strain after loading and unloading of one cyclic dynamic loading. As indicated in Fig. 6, the hysteresis curve of CGSF under cyclic loading is unclosed and asymmetric, which is due to the unrecoverable plastic deformation caused by cyclic loading for coal gangue sample. In addition, the center of hysteresis curve gradually moves towards to the increasing direction of dynamic strain, which shows the behavior of gradually accumulated strain.

**Figure 8:**
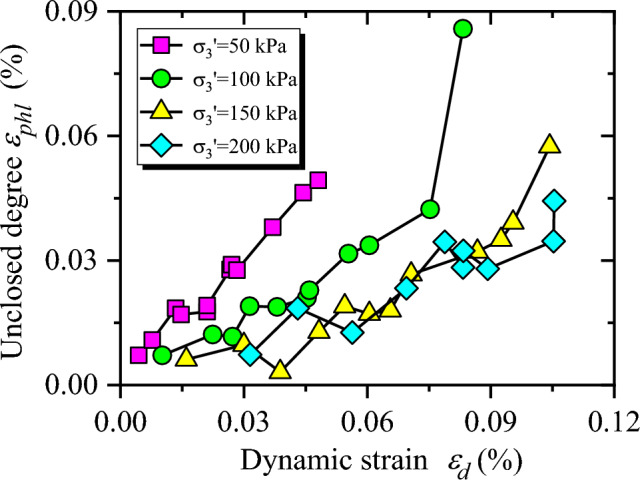
The relationship between $\varepsilon_{phl}$ and $\varepsilon_{d}$.

According to Eq. (8), the unclosed degree ($\varepsilon_{phl}$) of hysteresis curve can be captured. The relationship between $\varepsilon_{phl}$ and dynamic strain ($\varepsilon_{d}$) is presented in Fig. 8. As demonstrated in Fig. 8, (1) With the increase of dynamic strain, the unclosed degree of hysteresis curve increases exponentially. It shows that the bigger residual deformation of CGSF occurs with the larger amplitude of dynamic stress. (2) As the increasing confining pressure, the unclosed degree of hysteresis curve decreases. The results show that the compaction effect of confining pressure on CGSF leads to the interlocking between coal gangue particle–particle enhancement, and the cohesion and friction force increase, which causes the residual deformation difficult to develop. Therefore, confining pressure has an obvious inhibitory effect on the development of unclosed degree. Zhuang et al.^37^ studied the MCHC of improved expansive soil by using cylinder samples with a diameter of 50 mm and a height of 100 mm. However, due to the dynamic characteristic test including coal gangue coarse particles carried out in this test, the coarse particles will slip and break during the loading process, resulting in the hysteresis curve not smooth enough and some fluctuations in the curve.

### Analysis of the inclination of long axis degree

As demonstrated in Fig. 6, the hysteresis curve of CGSF under cyclic loading is asymmetric in the process of loading and unloading, and the hysteresis curve is unclosed. The gradient of the straight line through two points, i.e., the turning point of the loading and unloading (such as point $D_{l}$ in Fig. 7) and the midpoint of the starting and ending points of the loading and unloading (such as point $C_{l}$ in Fig. 7) in one cyclic loading, is used to characterize the inclination of the long axis ($k_{hl}$) of the hysteresis curve. Then, the inclination of the long axis ($k_{hl}$) of the hysteresis curve is defined as

9 $$k_{hl} = \frac{{\sigma_{D} - \sigma_{C} }}{{\varepsilon_{D} - \varepsilon_{C} }}$$

where $k_{hl}$ denotes the inclination of the long axis of the hysteresis curve, $\varepsilon_{D}$ and $\sigma_{D}$ represent the dynamic strain and dynamic stress at the turning point of loading and unloading in one cycle, respectively, $\varepsilon_{C}$ and $\sigma_{C}$ is the dynamic strain and dynamic stress at the midpoint of the starting and ending points of the loading and unloading in one cycle, respectively.

$k_{hl}$ reflects the stiffness and elastic properties of CGSF. The greater $k_{hl}$ has the better elastic properties of CGSF and the greater dynamic elastic modulus. On the contrary, the lower dynamic elastic modulus occurs with the smaller $k_{hl}$, and the specimen is easy to soften.

**Figure 9:**
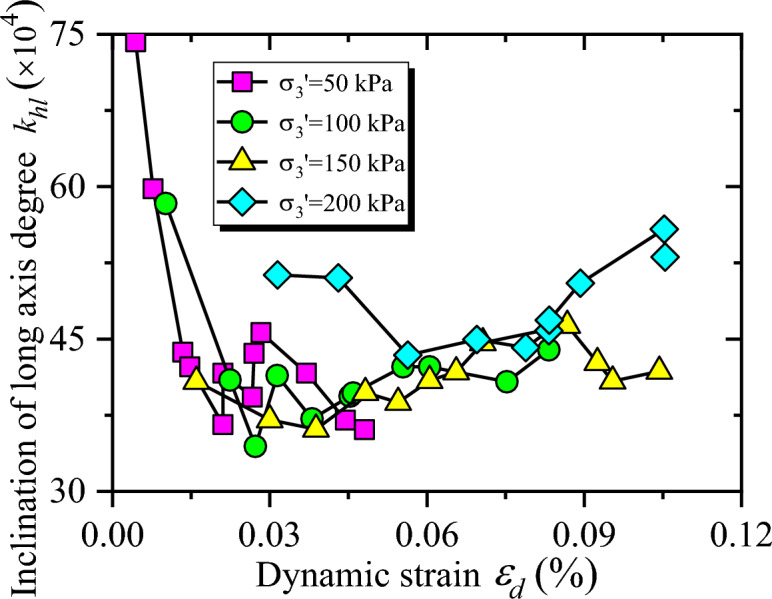
The relationship between $k_{hl}$ and $\varepsilon_{d}$.

Relationships between $k_{hl}$ and $\varepsilon_{d}$ are displayed in Fig. 9. Obviously, $k_{hl}$ of the coal gangue sample first decreases and then shows an increasing trend with the increasing dynamic strain. This shows that the resistance to deformation of the specimen is strong at the initial stage of step loading. Furthermore, with the increase of dynamic stress, the resistance to deformation of the specimen gradually decreases and slightly strengthened at the later stage of step loading. On the other hand, with the increase of confining pressure, $k_{hl}$ of the hysteresis curve shows a gradually increasing trend, which means that the dynamic elastic modulus raises with the increasing confining pressure. With the increase of confining pressure, the initial stress of the coal gangue sample increases, the void ratio reduces, the cohesion and friction of the sample raise. Then the sliding between each particles becomes difficult, which improves the ability of specimen to resist deformation.

### Analysis of the area for hysteretic curve

Because the hysteresis curve is unclosed, the starting and ending points of the loading and unloading in one cycle is connected in this paper, and then the area of the closed hysteresis curve is solved. Firstly, the hysteresis curve is artificially divided into two sections, i.e., the gradient is positive or negative. Then the area enclosed by the hysteresis curve with negative gradient and the abscissa axis is subtracted from the area enclosed by the hysteresis curve with positive gradient and the abscissa axis to calculate the area of the hysteresis curve, and the calculation formula is

10 $$S_{hl} = \frac{1}{2}\mathop \sum \limits_{i = 1}^{n} \left( {\sigma_{i} + \sigma_{i + 1} } \right)\left( {\varepsilon_{i + 1} - \varepsilon_{i} } \right) - \frac{1}{2}\mathop \sum \limits_{j = 1}^{m} \left( {\sigma_{j} + \sigma_{j + 1} } \right)\left( {\varepsilon_{j + 1} - \varepsilon_{j} } \right)$$

where $S_{hl}$ presents the area of the hysteresis curve; $\varepsilon_{i}$ and $\sigma_{i}$ are the dynamic strain and dynamic stress at each point in the hysteresis curve with positive gradient, respectively; $\varepsilon_{i}$ and $\sigma_{i}$ indicate the dynamic strain and dynamic stress at each point in the hysteresis curve with negative gradient, separately.

The area of hysteresis curve reflects the energy dissipation of coal gangue sample due to damping. The larger area of the hysteresis curve has the greater energy dissipation of the sample under one cyclic loading.

**Figure 10:**
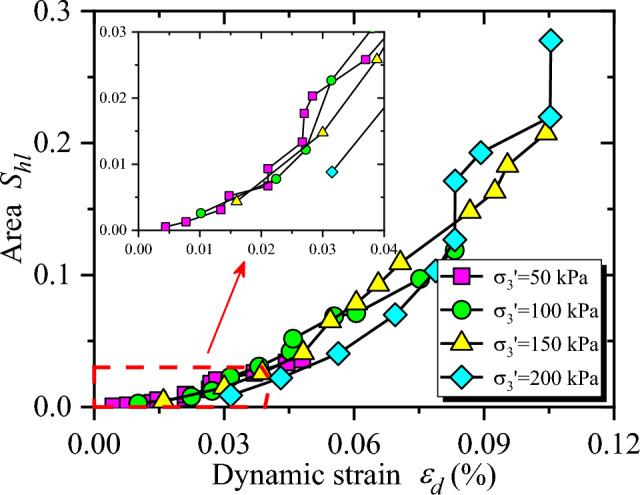
The relationship between $S_{hl}$ and $\varepsilon_{d}$.

$S_{hl}$ of CGSF can be obtained by Eq. (10), and the evolution of $S_{hl}$ versus $\varepsilon_{d}$ is illustrated in Fig. 10. As shown in Fig. 10, the curves are intertwined and difficult to distinguish under the condition of small strain. Therefore, the embedded diagram is displayed in Fig. 10 to show the curve variation with dynamic strain ($\varepsilon_{d}$) less than 0.04%. It can be seen from Fig. 10 that the sample can rebound sufficiently when the dynamic strain is small ($\varepsilon_{d} < 0.04 \%$). Hence, the values of $S_{hl}$ are close to each other, and the energy dissipation in the sample is small. However, with the increase of dynamic strain, the sample is gradually compressed. Then, the specimen failure degree is increased, $S_{hl}$ increases exponentially, and the damping ratio increases. With the increasing confining pressure, $S_{hl}$ shows a decreasing trend.

### Analysis of the fullness degree

As observed in Fig. 6, the fullness degree of hysteresis curve is various under different test conditions. Hence, $\alpha_{hl}$ is used to analyze the fullness degree of hysteresis curve, which is defined as

11 $$\alpha_{hl} = \frac{{b_{hl} }}{{a_{hl} }}$$

where $\alpha_{hl}$ presents the fullness degree; $b_{hl}$ is the length of the long axis for the hysteresis curve, i.e., the distance between point $C_{l}$ and point $D_{l}$ in Fig. 7; $a_{hl}$ represents the length of the short axis for the hysteresis curve, considering that the loading and unloading of the hysteresis curve as shown in Fig. 6 is asymmetric and not smooth, the sum of the line segment $E_{l} F_{l}$ and $F_{l} G_{l}$ is taken as $a_{hl}$, i.e., the sum of the maximum distance between the loading and unloading section of the hysteresis curve and the straight line $C_{l} D_{l}$.

$\alpha_{hl}$ reflects the viscosity of CGSF, the greater $\alpha_{hl}$ has the bigger viscosity. In the hysteresis curve of CGSF shown in Fig. 6, the thinner hysteresis curve occurs with the smaller $\alpha_{hl}$, and the fatter hysteresis curve indicates the larger $\alpha_{hl}$.

**Figure 11:**
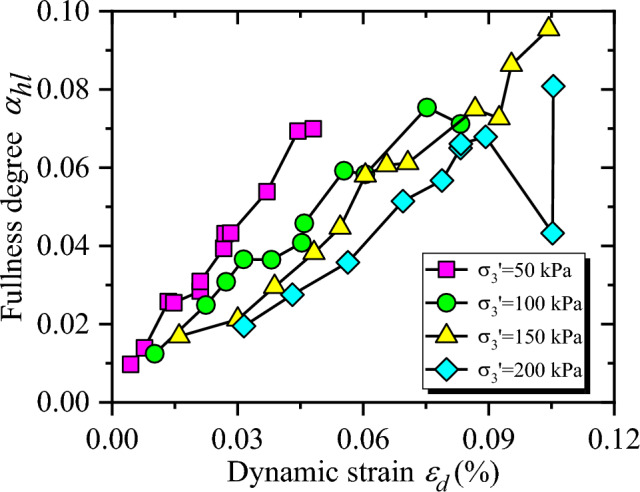
The relationship between $\alpha_{hl}$ and $\varepsilon_{d}$.

Relationships among $\alpha_{hl}$ and $\varepsilon_{d}$ are illustrated in Fig. 11, with the increase of dynamic strain, $\alpha_{hl}$ increases approximately linear, and the influence of confining pressure on $\alpha_{hl}$ is significant, that is, the greater the confining pressure is, the smaller the $\alpha_{hl}$ will be. Furtherly, with the increasing dynamic strain, particles of CGSF are gradually compacted, and then particle sliding or even breakage occurs. The structure of the sample is failed, and the unrecoverable plastic deformation is gradually generated. Under cyclic loading, the viscosity of the sample increases, and the hysteresis curve becomes fuller. The larger confining pressure results in the closer contact between particle and particle, the more compact sample, the lower viscosity of sample under cyclic loading, and the thinner hysteresis curve. In this test, under the condition of small strain, the coarse particles of the sample were gradually compacted, and the elastic energy was not obvious enough, so there was no obvious turning point in the test results.

## Conclusions

Hysteresis curve morphological characteristics can characterize the important dynamic behaviors of the specimen, such as dynamic deformation, viscosity, stiffness, energy loss, dynamic modulus, damping ratio. Therefore, the study of dynamic characteristics of coal gangue subgrade filler using the hysteretic curves is of great significance for understanding the dynamic behaviors of actual subgrade engineering under traffic load. Based on the large-scale dynamic triaxial tests of coal gangue subgrade filler, the following conclusions can be drawn from the above investigation.

1. The hysteresis loop of CGSF under cyclic loading is approximately oval and not smooth. The detailed calculation method of MCHC parameters was proposed, and then the MCHC parameters were used to quantitatively investigate the evolution of MCHC of CGSF.
2. With the increase of dynamic strain, $\varepsilon_{phl}$ increases exponentially. Confining pressure has an obvious inhibitory effect on the development of $\varepsilon_{phl}$, as the increasing confining pressure, $\varepsilon_{phl}$ decreases.
3. $k_{hl}$ of the coal gangue sample first decreases and then shows an increasing trend with the increasing dynamic strain. Furthermore, with the increase of dynamic stress, the resistance to deformation of the specimen gradually decreases and slightly strengthened at the later stage of step loading. On the other hand, with the increase of confining pressure, $k_{hl}$ shows a gradually increasing trend, which means that the dynamic elastic modulus raises with the increasing confining pressure.
4. The values of $S_{hl}$ are close to each other, and the energy dissipation in the sample is small. However, with the increase of dynamic strain, the specimen failure degree is increased, $S_{hl}$ increases exponentially, and the damping ratio increases. With the increasing confining pressure, $S_{hl}$ shows a decreasing trend.
5. With the increase of dynamic strain, $\alpha_{hl}$ increases approximately linearly, and the influence of confining pressure on $\alpha_{hl}$ is significant, that is, the greater the confining pressure is, the smaller the $\alpha_{hl}$ will be.

## Data Availability

The datasets used and/or analysed during the current study available from the corresponding author on reasonable request.
